# Acute Pericarditis Post mRNA-1273 COVID Vaccine Booster

**DOI:** 10.7759/cureus.22148

**Published:** 2022-02-12

**Authors:** Arminder Singh, Lam Nguyen, Stephanie Everest, Safi Afzal, Ahmed Shim

**Affiliations:** 1 Internal Medicine, Cape Fear Valley Medical Center, Fayetteville, USA; 2 School of Medicine, Campbell University School of Osteopathic Medicine, Lillington, USA; 3 Electrophysiology, Cape Fear Valley Medical Center, Fayetteville, USA

**Keywords:** covid-19, pericarditis, mrna-1273 vaccine, booster side effects, booster vaccine

## Abstract

Cardiovascular complications such as arrhythmias, hypoxemic cardiomyopathy, pericarditis, myocardial infarction, heart failure, and myocarditis are rare but seen in COVID-19 patients. These cardiac injuries could be the result of direct SARS-CoV-2 effects. The most prominent mediator of this hypothesis is angiotensin-converting enzyme-2 (ACE2) receptors, which are highly expressed in heart and lung tissues. These ACE2 receptors are found to be the functional receptors for the Coronavirus. Another hypothesis for cardiac complications in COVID-19 patients is macrophage-induced inflammation. The SARS-CoV-2 infection leads to invasion of epithelial cells by binding with ACE-2 receptors, localized inflammation, endothelial and macrophage activation, tissue damage, and dysregulated cytokine release. Current data have shown that mRNA COVID-19 vaccines are efficacious and safe for indicated patients. However, these vaccines can cause mild adverse reactions similar to those of traditional vaccines, and more severe side effects can also be seen infrequently. The exact pathogenesis of COVID-19 vaccine-induced pericarditis remains unknown, but there are several hypotheses regarding the pathophysiology of pericarditis after COVID-19 vaccine administrations. There has been speculation that mRNA vaccines can produce a large number of antibodies in a small subgroup of people, especially young individuals, and this elicits an inflammatory response similar to the multisystem inflammatory syndrome associated with SARS-CoV-2 infection. Another proposed mechanism is the cross-reaction between produced antibodies and the pericardium, leading to myocardial and pericardial inflammation induction.

This report describes a 69-year-old female who presented with three days of chest pain that started one day after a booster shot of the Moderna COVID-19 vaccine. The patient was diagnosed with pericarditis, and she was effectively treated with colchicine and later steroids.

## Introduction

In the last couple of years, several Coronavirus disease 2019 (COVID-19) vaccines have been developed and authorized for human use to prevent infections or reduce the severity and complications of COVID-19. Of these vaccines, mRNA-1273 by Moderna and BNT162b2 by Pfizer were the first vaccines to receive emergency use authorization by the United States Food and Drug Administration (FDA). They are also two of the first mRNA vaccinations widely used in humans [1]. Thus far, these mRNA COVID-19 vaccines have excellent efficacy and safety profiles in clinical trials and population-wide use. Similar to other traditional vaccines, mRNA-1732 and BNT162b2 can cause side effects [2]. The most common adverse effects include erythema, swelling, lymphadenopathy, injection site pain, fever, headache, fatigue, myalgia, or arthralgias. The majority of these reactions are usually mild. In addition, there are also reports of more severe side effects such as thromboembolic events, myocarditis, and pericarditis [2-4]. However, myocarditis or pericarditis following vaccinations is still considered rare. As of December 16, 2021, more than 470 million doses of mRNA COVID-19 vaccinations have been administered in the U.S. In addition, the CDC has received 1,947 preliminary reports and verified 1,124 cases of myocarditis or pericarditis following vaccination [5].

In this case report, we report a 69-year-old female who presented with three days of worsening chest pain four days after receiving the mRNA-1273 vaccine booster and was subsequently diagnosed with pericarditis. We also discuss the current literature surrounding the incidence, presentation, and management of myocarditis.

## Case presentation

**Figure 1:**
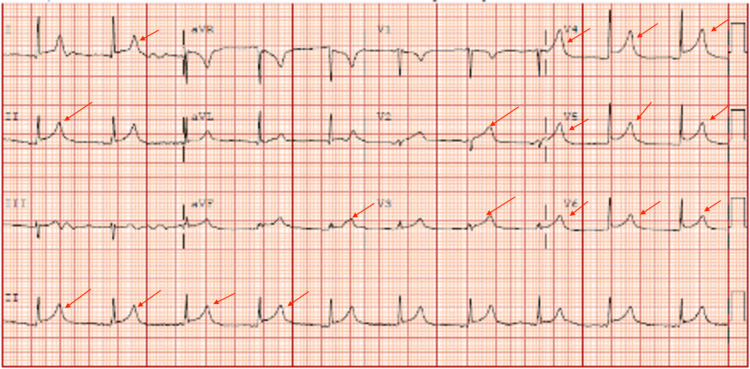
Electrocardiogram demonstrating diffuse ST-segment elevations Red arrows demonstrating the ST-segment elevations

A 69-year-old female with a history of morbid obesity, hypertension, diabetes, gout disease, and chronic kidney disease stage 3b with an EGFR of 30.2 mL/min/1.73 m^2^ presented to the hospital with a three-day history of worsening chest pain. It was a crushing chest pain that radiated to the base of her neck. The pain was aggravated when she lay flat. Of note, the patient had received her second dose of Moderna COVID-19 vaccine one day before symptoms onset. Initial labs were significant for unremarkable troponin and blood urea nitrogen (BUN)/creatinine of 34/1.51. The BUN/creatinine level was within the baseline for the patient. She tested negative for SARS-CoV-2 PCR, influenza A and B PCR, and respiratory syncytial virus (RSV) PCR. She also denied any viral-like symptoms before the onset of her chest pain. An electrocardiogram (ECG) showed diffuse ST-segment elevation (Figure 1). The C-reactive protein (CRP) was over 190 mg/L, and the sedimentation rate (ESR) was over 130 mm/hr.

**Figure 2:**
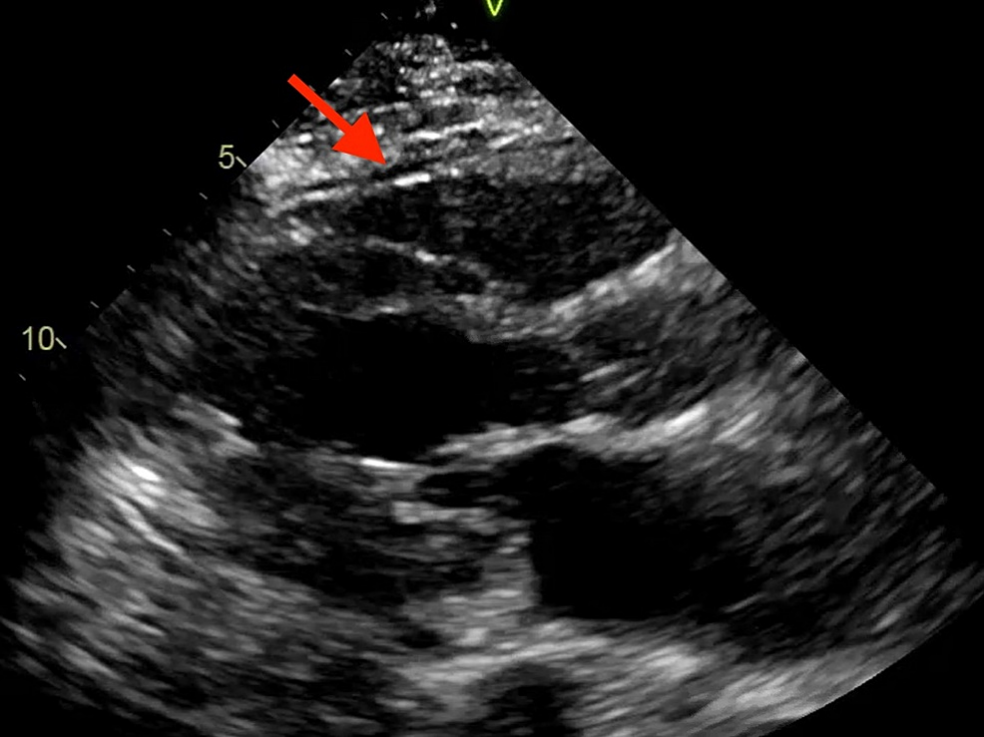
Transthoracic echocardiogram demonstrating small pericardial effusion Red arrow demonstrating the small pericardial effusion

**Figure 3:**
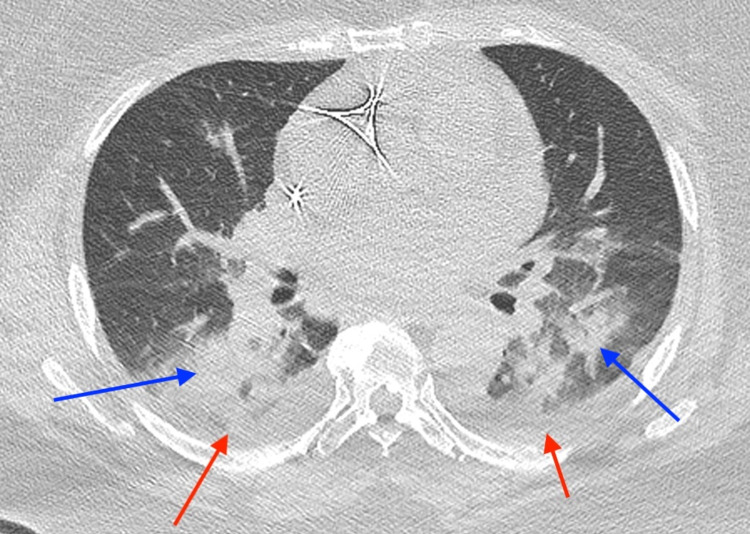
Computed tomography without contrast demonstrating small bilateral pleural effusions and infiltrates Red arrows: demonstrating the bilateral pleural effusions; blue arrows: demonstrating the bilateral infiltrates

With concerns of ST-segment elevation myocardial infarction (STEMI), emergent cardiac catheterization was performed, revealing no evidence of obstructive coronary artery disease. A transthoracic echocardiogram (TTE) was also performed, demonstrating normal left ventricular systolic function with an ejection fraction greater than 55%. The TTE revealed a small pericardial effusion without any obvious pericardial inflammation (Figure 2). Therefore, the patient was diagnosed with pericarditis secondary to COVID vaccine administration. Infectious etiologies were low on the differentials as the patient remained afebrile with a normal white blood count and was negative for two sets of blood cultures. Also, autoimmune etiologies were low on the differential as the patient tested negative for antinuclear antibodies (ANA), anti-double-stranded antibodies (anti-dsDNA), and anti-neutrophilic cytoplasmic antibodies (PANCA). During her hospital stay, she developed paroxysmal atrial fibrillation and tachycardic-bradycardic syndrome with frequent significant pauses on telemetry that required dual-chamber placement. The patient was treated with oral colchicine renal dosage of 0.3 mg a day, as non-steroidal anti-inflammatory drugs were not initiated due to decreased renal function on repeat BUN/Cr labs. However, colchicine was discontinued on day 4 of treatment due to adverse effects of diarrhea, and intravenous methylprednisolone 40 mg daily was started as an alternative. Additionally, a chest computed tomography without contrast was performed on day 4 of the admission due to the patient’s complaints of mild shortness of breath, which showed diffuse bilateral infiltrates and pleural effusion with a small pericardial effusion without any evidence of pericardial inflammation (Figure 3). The patient’s pulmonary findings were also thought to be caused by an inflammatory reaction from the COVID-19 vaccine, so methylprednisolone was continued as its treatment. The patient was discharged in stable condition three days after the steroid initiation, and her treatment was transitioned to oral prednisone for another four weeks prior to a cardiology follow-up.

## Discussion

Infection with COVID-19 has been shown to significantly increase the risk of cardiovascular complications, including myocardial injury and myocarditis, acute myocardial infarction, heart failure and cardiomyopathy, arrhythmias, shock, cardiac arrest, and venous thromboembolic events [6]. For example, a recent study examining data obtained from a large hospital-based administrative database found that rates of myocarditis were 16 times higher in patients infected with COVID-19 [7]. In addition, an increased risk of myocarditis and pericarditis has been reported following mRNA vaccinations, particularly in adolescent males. A September 2021 systematic review of the literature analyzed 69 available case reports and case series of cardiac complications following mRNA vaccinations and found that out of 243 patients, 227 were diagnosed with myocarditis/myopericarditis, and seven were diagnosed with pericarditis. The majority (92%) were males, and the mean age was 21 years (range, 12-70 years). Three-quarters of the patients who were diagnosed with myocarditis or pericarditis had received the BNT162b2 vaccine (Pfizer-BioNTech), and most (88%) were diagnosed after the second vaccine dose, with a median time from immunization to symptom onset of three days (range: 1.5 hours to 73 days) [8].

After the publication of the COVID-19 causative organism's complete genome, the severe acute respiratory syndrome coronavirus 2 (SARS-CoV-2) by Zhang and Holmes in January 2020, the race to develop its vaccines has happened at an unprecedented pace [1]. In a matter of months, mRNA-1273 by Moderna was the first COVID-19 vaccine to enter clinical trials in the U.S. The mRNA-1273 sequence encodes for the pre-fusion form of S protein, a SARS-CoV-2 surface protein that binds to host angiotensin-converting enzyme receptors, leading to viral fusion and entry into host cells. Upon administration of the mRNA-1273 vaccine, cellular production of S proteins is initiated with these mRNA templates, and the host immune system starts to make antibodies to these antigenic S proteins as a result. The exact pathogenesis of COVID-19 vaccine-induced pericarditis remains unknown, but there are several hypotheses regarding the pathophysiology of pericarditis after COVID-19 vaccine administrations [3,4]. There has been speculation that mRNA vaccines can produce a large number of antibodies in a small subgroup of people, especially young individuals, and this elicits an inflammatory response similar to the multisystem inflammatory syndrome associated with SARS-CoV-2 infection. Another proposed mechanism is the cross-reaction between produced antibodies and the pericardium, leading to myocardial and pericardial inflammation induction. 

Pericarditis is inflammation of the pericardium, and it is the most common disorder of the pericardium. The most common causes of pericarditis include viral infections, bacterial infections, malignancy, rheumatoid arthritis, uremia, myxedema, or systemic lupus erythematosus [9]. The diagnosis of pericarditis can be made with clinical manifestation, echocardiography, electrocardiogram, lab findings, or other imaging modalities. Once diagnosed with pericarditis, patients can be adequately managed with medical therapy. High doses of nonsteroidal anti-inflammatory drugs (NSAIDs) such as naproxen, ibuprofen, or Indomethacin are effective first-line agents for pericarditis. Colchicine has been shown to significantly reduce recurrent episodes of pericarditis when used in combination with NSAIDs. Thus, patients with pericarditis are usually treated with colchicine and high-dose NSAIDs. Colchicine is commonly used for three to six months, and NSAIDs are utilized until symptom relief is achieved. In the event of NSAIDs and colchicine not being used, low to moderate doses of steroids can work as alternatives, but the risk of recurrence is higher after discontinuation of steroids.

Our patient presented with cardinal signs and symptoms of pericarditis, including chest pain, worsening when lying flat, normal troponin levels, and diffuse ST-segment elevation on ECG. Her symptoms started one day after receiving the second dose of the Moderna COVID-19 vaccine. Thus, her pericarditis was thought to be caused by the vaccine administration, and the pericarditis was diagnosed based on the patient's clinical symptoms, ECG changes, TTE findings, and CT results. NSAIDs were not started for the patient due to her renal function. Therefore, colchicine was used as an initial treatment for her. However, colchicine was discontinued due to diarrhea, and intravenous methylprednisolone was initiated as replacement therapy. Fortunately, the patient responded well to the steroids, and she was later discharged with a four-week maintenance dose of prednisone. The patient's favorable response to the therapy helps validate the current treatment guidelines for pericarditis, and it can also help guide the treatment approaches for pericarditis induced by mRNA COVID-19 vaccines.

## Conclusions

Pericarditis is a rare complication associated with mRNA COVID-19 vaccine administration. Further studies are needed to understand the pathogenesis of the disease process fully. Yet, our patient’s positive response to the treatment would help to guide future therapy for a similar patient population. Also, as the rate of mRNA COVID-19 vaccine pericarditis is rare among patients receiving the vaccines, we still highly recommend patients receive the COVID-19 vaccines due to their overwhelming benefits. 
